# Supplementary material for: Current-driven writing process in antiferromagnetic Mn2Au for memory applications
Source: Nat Commun. 2023 Apr 3;14:1861. doi: 10.1038/s41467-023-37569-8 (PMC10070341; doi:10.1038/s41467-023-37569-8)
Supplement: Supplementary file 1 — Supplemantary Information [file 41467_2023_37569_MOESM1_ESM.pdf]

SUPPLEMENTARY INFORMATION: Current-driven writing process in antiferromagnetic Mn_2Au for memory applications

S. Reimers †,[1] . Lytvynenko †,[1,2] Y. R. Niu,[3] E. Golias,[3] B. Sarpi,[4] L. S. I. Veiga,[4] T. Denneulin,[5] A. Kovács,[5] R. E. Dunin-Borkowski,[5] M. Kläui,[1] and M. Jourdan*[1]

[1] *Institut für Physik, Johannes Gutenberg-Universität Mainz, 55099 Mainz, Germany*
[2] *Institute of Magnetism of the NAS of Ukraine and MES of Ukraine, 03142 Kyiv, Ukraine*
[3] *MAX IV Laboratory, Fotongatan 8, 22484 Lund, Sweden*
[4] *Diamond Light Source, Chilton, Didcot, Oxfordshire, OX11 0DE, United Kingdom*
[5] *Ernst Ruska-Centre for Microscopy and Spectroscopy with Electrons, Forschungszentrum Jülich, 52425Jülich, Germany*

SUPPLEMENTARY INFORMATION

Current pulse induced sample heating

All current pulsing experiments are associated with ohmic heating of the sample. As Mn_2Au is a metal with an almost linear resistance versus temperature relation, the current pulse induced heating can be measured via a resistance measurement during the pulse as described in the Methods section. Supplementary Fig. S1 shows the relative resistance increase of a $Mn_2Au(001)$ sample patterned in a cross structure with a central section of 10 µm × 10 µm as in Fig. 1 of the main text, but with additional leads to enable 4-probe resistance measurements. Different pulse lengths and amplitudes result in different heating. The dashed vertical lines in the main figure indicate the required current densities for complete switching of the Néel vector. From comparison with the extrapolated temperature dependent resistance measurement shown in the inset, the indicated temperatures associated with the switching using different pulse lengths are shown in the main panel.

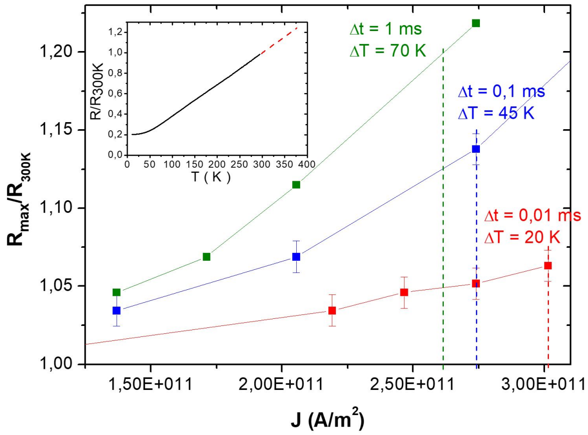

FIG. 1. **S1: Current pulse induced sample heating.** The measurements in the main panel show the maximum sample resistance (normalized to the 300 K resistance) during application of current pulses with different length and amplitude. The dashed vertical lines show the required currents for full Néel vector reorientation corresponding to Fig. 1. The inset shows the temperature dependence of the normalized resistance obtained from a standard dc measurement. Its extrapolation (red dashed line) is used to convert the resistance increase during current pulsing into a temperature increases ΔT shown in the graph.

Probing Néel vector reorientation by transverse resistance measurements

Electrical detection of current pulse induced Néel vector alignment is also possible via measurements of the transverse resistance or planar Hall-Effect. Considering the rather small electrical signal, this is more easy to measure than the longitudinal AMR, as there is no offset voltage. In Supplementary Fig. S2, the measurement of the transverse resistance changes is shown in analogy to Fig. 4 of the main text.

Stability of the Néel vector alignment

All images of current induced Néel vector reorientation shown in the main text were obtained at MAXPEEM (MAX IV). Four months later at Diamond Light Source, we were able to image the sample shown in Fig. 2 of the main text again. As shown in Supplementary Fig. S3, we observed exactly the same domain pattern again, i. e. the current induced Néel vector oriented domain configuration obtained four month ago was completely preserved.

Additionally, we demonstrate the absence of relaxation on short time scales, i. e. seconds, by measurements of the transverse resistance after a sequence of current pulse induced reversible switching. The data shown in Supplementary Fig. S4 was obtained in the same way as for Supplementary Fig. S2 (here with $J_{\mathrm{pulse}} = 2.9 \times 10^{11}$ A/m^2), but we kept measuring the resistance after the last switching current pulse for 1.5 h. The raw data shown was obtained at a sampling rate of $\simeq 2$ data points per second. Also on this time scale, no relaxation of the resistance after the last switching pulse was observed.

Microstructure of $Mn_2Au(001)$ thin films

All epitaxial Mn_2Au thin films used for this manuscript were prepared by sputtering as described in the Methods sections. Beyond their characterization shown in ref. [20], we here want to emphasize their long range crystallographic order by showing in Supplementary Fig. S5 scanning transmission electron microscopy images. Panel **a** with the highest magnifications shows the Mn_2Au lattice with atomic resolution (on top of this sample is a Permal-

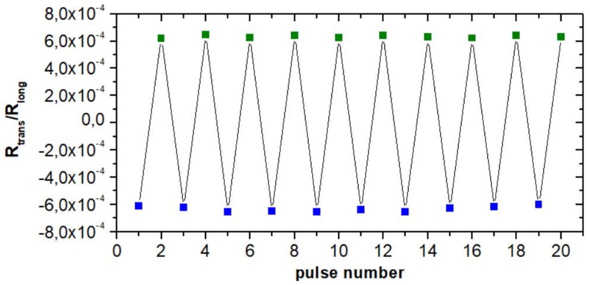

FIG. 2. **S2: Switching induced planar Hall effect.** The measurements shows the alternating transverse resistance of a Mn_2Au thin film patterned in the geometry shown in Fig. 4, panel **b**. The transverse resistance was measured after each 1 ms current pulse with $J_{\mathrm{pulse}} = 3 \times 10^{11}$ A/m^2 applied alternately in perpendicular directions as indicated by the blue and green arrows in Fig. 4, panel **b**. The color of the data points corresponds to the arrows indicating the current direction.

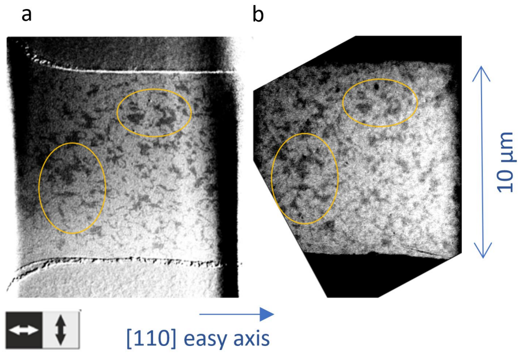

FIG. 3. **S3: Long term stability of switched state.** The XMLD-PEEM image in panel **a** is the same as shown in Fig. 2, right panel, obtained after current induces Néel vector reorientation. The image in panel **b** shows the same area 4 months later. The yellow circles serve to guide the eye showing exactly the same AFM domain pattern (please note that the image in panel **b** has reduced magnetic contrast due to a different PEEM geometry).

loy layer, which was not present in the other samples discussed here.). Panel **b** with a larger field of view shows a MnAu impurity phase at the interface with the Ta buffer layer, which, however, does not disturb the growth of the Mn_2Au layer on top of it. Panel **c** shows the largest field of view demonstrating the long range coherence of the epitaxial growth.

For obtaining the STEM images, an electron transparent cross-section lamella was prepared using a 30 kV focused Ga+ ion beam and scanning electron microscope (FIB-SEM) FEI Helios platform. The ion beam energy was decreased to 5 kV for the final thinning steps. Scanning transmission electron microscopy (STEM) was carried out using an FEI Titan TEM equipped with a Schottky field emission gun operated at 200 kV, a CEOS probe aberration corrector and a high angle annular dark-field

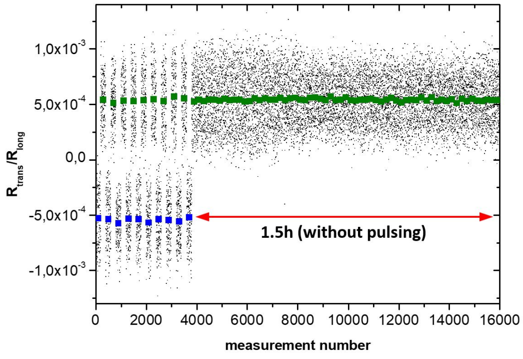

FIG. 4. **S4: Second time-scale stability of switched state.** Raw data (black data points) and averaged data points (blue and green data points as in Supplementary Fig. S2) over 200 raw data points (blue and green data points as in Supplementary Fig. S2) of the transverse resistance of a Mn_2Au thin film. Here, the transverse resistance was measured after each 1 ms current pulse with $J_{\mathrm{pulse}} = 2.9 \times 10^{11}$ A/m^2 applied alternately in perpendicular directions followed by pure resistance measurements without pulsing. No relaxation was observed.

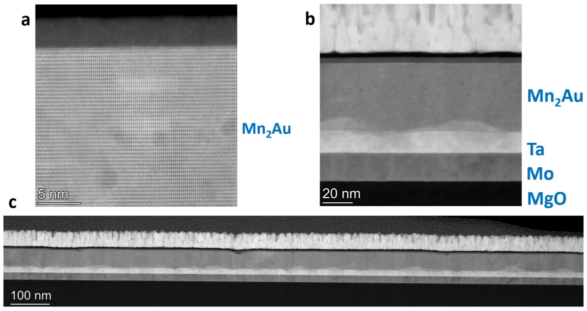

FIG. 5. **S5: Microstructure of a Mn_2Au(001) epitaxial thin film.** Cross section HAADF STEM images with different magnifications showing the microstructure of an epitaxial Mn_2Au(001) thin film. A weak MnAu impurity phase is visible at the interface between the Mn_2Au and the Ta buffer layer. The samples show long range crystallographic order.

detector (HAADF).
